# Thermodynamic and Kinetic Analysis of TiN Precipitation in Nickel-Based Superalloys During Solidification

**DOI:** 10.3390/ma17225443

**Published:** 2024-11-07

**Authors:** Jie Zhang, Chengwei Zeng, Haibin Zheng, Changlong Gu

**Affiliations:** College of Mechanical Engineering, Zhejiang University of Technology, Hangzhou 310014, China; 221122020320@zjut.edu.cn (C.Z.); 18858274591@163.com (H.Z.); 221123020460@zjut.edu.cn (C.G.)

**Keywords:** thermodynamics and kinetics, solidification, TiN precipitation, microsegregation, nickel-based superalloys

## Abstract

Large TiN inclusions in nickel-based superalloys promote micropore formation, compromising the mechanical properties of the alloys. However, current research lacks a comprehensive coupled model that considers both solute element microsegregation and TiN precipitation specifically in nickel-based superalloys. This study investigated TiN precipitation during the solidification of IN718 alloys through a combined thermodynamic and kinetic approach. A modified Clyne–Kurz model was applied to account for multi-element microsegregation, enabling an integrated analysis of both microsegregation and precipitation processes. The results indicated that solute elements in the molten alloy segregated to varying degrees during solidification. At an initial nitrogen concentration of 25 ppm, TiN inclusions precipitated when the solid fraction reached 0.256, eventually resulting in a total TiN precipitation of 64 ppm by the end of solidification, while residual nitrogen in the liquid phase decreased to 1.3 ppm. Increasing the initial nitrogen concentration from 10 ppm to 40 ppm advanced the onset of TiN precipitation and raised the total amount from 16 ppm to 126 ppm. Further analysis indicated that cooling rates of 0.03 °C/s, 0.06 °C/s, and 0.18 °C/s did not significantly affect the final TiN accumulation.

## 1. Introduction

The IN718 alloy is widely valued for its excellent high-temperature mechanical properties, corrosion resistance, and creep resistance, making it indispensable for high-performance applications in aerospace and other advanced industries [1]. However, to meet the stringent quality standards required in these fields, further refinement of the alloy is necessary. Aerospace components have stringent fatigue resistance requirements that are highly sensitive to the presence and level of inclusions within the alloy. Therefore, precise control over the size and quantity of inclusions is crucial for improving alloy quality.

Compared to oxide inclusions, TiN inclusions in nickel-based alloys are harder and more brittle, significantly affecting the alloy’s fatigue life. Studies show that nitride inclusions around 6 μm in size have a comparable impact on fatigue life to oxide inclusions as large as 25 μm [2]. During alloy solidification, differences in the solubility of titanium and nitrogen between the solid and liquid phases lead to their redistribution at the solid–liquid interface, resulting in the enrichment of these elements. When the actual concentration of Ti and N in the liquid phase surpasses the equilibrium limit, primary nitrides (primarily TiN) begin to precipitate, often nucleating on oxides; subsequently, primary carbides may precipitate on the surface of these TiN particles through heterogeneous nucleation [3,4]. These TiN particles may aggregate into clusters or form complex carbonitrides with primary carbides. Under applied stress, microcracks can initiate at these larger inclusions, ultimately leading to fatigue failure of the alloy. Furthermore, blocky carbonitrides can obstruct interdendritic channels, limiting the flow and feedability of the molten alloy, which results in higher porosity within the alloy ingot and a notable reduction in overall alloy quality [5].

Over the past few decades, controlling TiN inclusions in IN718 alloys has been a primary research focus. Young and Mitchell [6] provided a comprehensive analysis of nitrogen additions and removals, highlighting that TiN primarily precipitates in interdendritic regions toward the end of solidification, where it contributes to micropore formation that diminishes the alloy’s mechanical properties. Mitchell and Wang [7] investigated the solidification behavior and secondary phase precipitation in IN718. Their findings revealed that, as solidification progresses, NbC nucleates on pre-existing TiN particles. Depending on the C and N ratio in the alloy, these NbC particles may evolve into mixed (Nb + Ti) carbonitrides at later stages, which in turn alters the microstructure of the alloy. Sindhura et al. [8] further emphasized the importance of controlling nitrogen and titanium concentrations to limit undesirable TiN inclusions. Such inclusions cause microstructural issues like agglomeration and microporosity, ultimately weakening the alloy’s mechanical performance. Leonardo et al. [3] introduced a method to minimize TiN precipitation through the VIM-VAR process, which effectively eliminates nitrides and produces TiN-free IN718 alloys. This technique helps prevent micropore formation and grain boundary weakening caused by TiN inclusions. Overall, in IN718 alloys, TiN particles not only act as heterogeneous nucleation sites that facilitate the formation of NbC particles but also cluster with other inclusions. These precipitates exhibit poor distribution uniformity within the structure and are difficult to improve through heat treatment or homogenization, ultimately affecting the alloy’s microstructure and mechanical properties. The initial nitrogen concentration in the alloy significantly determines the timing and total amount of TiN precipitation. Therefore, controlling nitrogen content is crucial for minimizing TiN precipitation and enhancing the overall performance of the alloy.

Various microsegregation models have been developed to improve predictions of solute element redistribution between solid and liquid phases during alloy solidification. The Lever-Rule (L-R) model predicts solute distribution under the assumption of complete solute diffusion in both phases. In contrast, the Scheil model assumes complete diffusion in the liquid phase but none in the solid phase. However, more accurate predictions require accounting for the finite solute diffusion within the solid phase. The Brody–Fleming (B-F) model, introduced by Brody and Fleming [9], incorporates a back diffusion coefficient to address this need. Subsequent refinements to this coefficient were made by Clyne and Kurz [10], Ohnaka [11], Voller and Beckermann [12], and Won and Thomas [13]. Despite their advancements, these foundational models overlook the impact of inclusion precipitation on solute element microsegregation.

In fact, there is a strong interaction between the precipitation of inclusions and element microsegregation [14]. During solidification, solute element microsegregation enriches alloying elements in the residual liquid phase, promoting inclusion precipitation, which then depletes certain solutes from the system. This interaction highlights the importance of considering inclusion precipitation when predicting solute microsegregation. To address the issue of the interaction between inclusion precipitation and elemental microsegregation, several coupled models have been developed, integrating solute microsegregation and inclusion precipitation modules. For example, Yang et al. [15] used a coupled Scheil-based model to simulate solute segregation and TiN inclusion behavior during the directional solidification of the K418 alloy. Similarly, Que et al. [2] applied the Voller–Beckermann model (V-B model) alongside TiN precipitation and growth models to study TiN behavior during the continuous casting of Ti micro-alloyed steels. Liu and Gui et al. [16,17] employed the Clyne–Kurz model (C-K model) to predict TiN inclusions in steel, analyzing the effects of Ti and N content and cooling rates on TiN formation. Lee and Li et al. [18,19] employed the model based on Ohnaka’s theory, incorporating the kinetics of inclusion precipitation and growth to predict the behavior of TiN inclusions during solidification. Similarly, Shu et al. [20] combined a modified Ohnaka-based model with a KWM model to predict inclusion size distributions during steel solidification. Won and Thomas [13] modified the Ohnaka parameters and, combined with the V-B model, extended the cell crystal growth model for the application to columnar crystals. Despite these advancements, most research has concentrated on inclusion behavior in steels, leaving a gap in studies on element microsegregation and inclusion precipitation during the solidification of nickel-based superalloys.

In this paper, the solidification process of the IN718 molten alloy during vacuum arc remelting was studied. A coupled model was developed and implemented using the modified C-K model proposed by Won and Thomas to integrate solute element microsegregation with a TiN precipitation prediction. Additionally, a thermodynamic analysis was used to evaluate the conditions for TiN inclusion formation. By using the coupling model, we evaluated the extent of element microsegregation and TiN precipitation levels. The effects of varying element concentrations and local cooling rates on TiN formation were explored. This study aims to provide a theoretical foundation for future research on the solidification behavior of IN718 alloys.

## 2. Thermodynamic and Kinetic Analysis of TiN Inclusion Formation in IN718

This section systematically examined the conditions for TiN inclusion precipitation in IN718 ingots during vacuum arc remelting (VAR), based on thermodynamic and kinetic models. A microsegregation model was applied to calculate the segregation levels of titanium and nitrogen in the residual liquid phase throughout solidification process. The coupled model predicted the amount of TiN precipitation at each stage, as well as the cumulative precipitation over the entire solidification process. Additionally, the impact of the microsegregation of various elements on the precipitation timing, size, and quantity of TiN inclusions was examined.

### 2.1. The Features of TiN Inclusions at Different Locations

**Figure 1:**
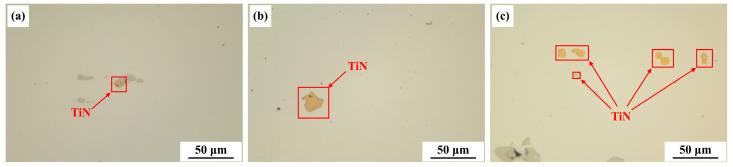
Features of TiN inclusions at various locations: (**a**) edge; (**b**) 1/2 radius; and (**c**) center.

Experiments were conducted on the ingot prepared by vacuum arc remelting (VAR), with metallographic samples taken from the edge, mid-radius, and center of the ingot. After grinding and polishing, the samples were examined under a metallographic microscope without corrosion treatment. Figure 1 shows the features of TiN inclusions at various locations within the ingot.

These images reveal that TiN inclusions are primarily block-shaped and generally smaller than 25 μm. At the ingot edge, TiN inclusions are relatively small, around 8 μm. As the distance to the center decreases, both the size and quantity of TiN inclusions increase. The trend can be attributed to the slower cooling rates that lead to larger secondary dendrite arm spacing near the ingot center, which, in turn, facilitated the precipitation and growth of TiN.

**Figure 2:**
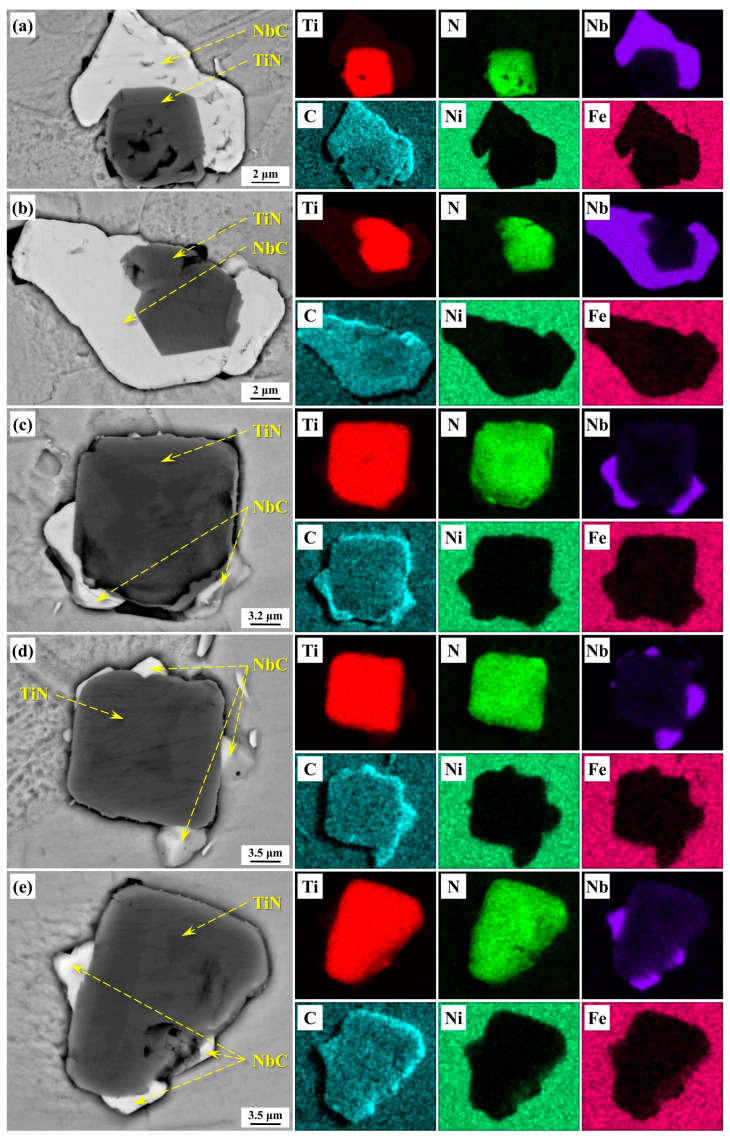
The SEM images and corresponding element mappings of typical nitride particles in the IN718 alloy: (**a**) nitride particle #1; (**b**) nitride particle #2; (**c**) nitride particle #3; (**d**) nitride particle #4; (**e**) nitride particle #5.

**Figure 3:**
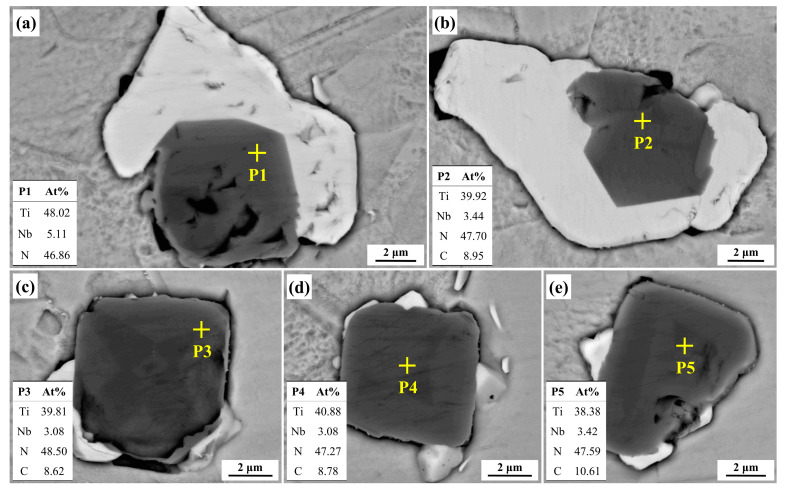
The results of point analyses for typical nitride particles in IN718 alloy: (**a**) nitride particle #1; (**b**) nitride particle #2; (**c**) nitride particle #3; (**d**) nitride particle #4; (**e**) nitride particle #5.

In the IN718 alloy, TiN and NbC share similar crystal structures, making some degree of niobium and carbon incorporation in TiN particles unavoidable. To determine the elemental composition of TiN particles, we conducted scanning electron microscopy (SEM) observations on several representative TiN particles in the alloy samples. The SEM results, shown in Figure 2, indicated that Ti and N were the primary components in TiN particles, though small amounts of C and Nb were also present. For a more detailed elemental analysis, semi-quantitative point analyses were performed on the TiN particles. Figure 3 shows the locations and results of these analyses, revealing that Ti and N contents in the analyzed areas are approximately 38–48% and 46–49%, respectively, while Nb and C contents are only about 3–5% and 8–10%. The Ti-to-Nb ratio is approximately 13:1, and the N-to-C ratio is around 5:1. Thus, TiN particles are composed primarily of Ti and N, containing extremely low levels of Nb and C. Given the low Nb and C levels, their effects on TiN particles were neglected in this study, treating TiN as pure TiN for calculation purposes. The thermodynamic conditions for TiN formation are discussed in the following section.

### 2.2. Thermodynamic Analysis of TiN Precipitation Equilibrium

The chemical composition of the IN718 ingot used in this study is presented in Table 1. A thermodynamic analysis was conducted to examine the precipitation process of TiN inclusions, with the relevant chemical reaction equation and precipitation conditions provided below [21].

(1) $$[\mathrm{Ti}]+[N]=\mathrm{TiN}(s)\;\Delta G^{Ө}=-379000+149T$$

(2) ΔG≤0

where Δ*G*^Ө^ is the standard Gibbs free energy, J/mol; Δ*G* is the Gibbs free energy of the reaction, J/mol; and *T* is the temperature, K.

According to the definition of Gibbs free energy, the equation for Δ*G* is given as follows:

(3) $$\Delta G=\Delta G^{Ө}+RT\ln\frac{a_{\mathrm{TiN}}}{f_{\mathrm{Ti}}\omega {\left[\mathrm{Ti}\right]}_{\%}\cdot f_{N}\omega {\left[N\right]}_{\%}}$$

where *a*_TiN_ denotes the activity of TiN in the molten alloy. According to Henry’s law, *f*_Ti_ and *f*_N_ represent the activity coefficients of Ti and N in molten alloy, respectively, with 1% concentration as the standard; *ω*[Ti]_%_ and *ω*[N]_%_ represent the actual concentrations of Ti and N in the molten alloy, respectively; R is the ideal gas constant, 8.314 J·mol^−1^·K^−1^; for pure TiN, the activity is 1. Then, Equation (3) is rewritten as follows:

(4) $$\begin{array}{cc} \Delta G & =\Delta G^{Ө}-RT\left(\ln f_{\mathrm{Ti}}+\ln f_{N}+\ln\omega {\left[\mathrm{Ti}\right]}_{\%}\cdot \omega {\left[N\right]}_{\%}\right)\\ & =\Delta G^{Ө}-\frac{RT}{\mathrm{lg}e}(\mathrm{lg}f_{\mathrm{Ti}}+\mathrm{lg}f_{N}+\mathrm{lg}\omega {\left[\mathrm{Ti}\right]}_{\%}\cdot \omega {\left[N\right]}_{\%}) \end{array}$$

The natural constant e ≈ 2.718; while lg*f*_Ti_ and lg*f*_N_ can be determined using the activity interaction equations:

(5) $$\mathrm{lg}f_{i}=\sum \left(e_{i}^{j}\cdot [\mathrm{pct}j]+r_{i}^{j}\cdot {\left[\mathrm{pct}j\right]}^{2}\right)$$

where $e_{i}^{j}$ and $r_{i}^{j}$ represent the first-order and second-order activity interaction coefficients of solute element *j* with element *i* in the molten alloy, and [pct*j*] denotes the actual concentration of element *j*. The chemical composition of the IN718 alloy used in this study is presented in Table 1. Table 2 lists the first-order and second-order activity interaction coefficients used in this study’s thermodynamic calculations.

From Equation (5), values for lg*f*_Ti_ ≈ 0.487 and lg*f*_N_ ≈ −2.314 are obtained. Subsequently, an expression for the concentration product of Ti and N is derived.

(6) $$\mathrm{lg}(\omega {\left[\mathrm{Ti}\right]}_{\%}\cdot \omega {\left[N\right]}_{\%})=\frac{\mathrm{lg}e\cdot (\Delta G^{Ө}-\Delta G)}{RT}+1.827$$

When Δ*G* = 0, TiN precipitates in the liquid phase, and the equilibrium concentration product for TiN precipitation can be determined.

(7) $$\mathrm{lg}(\omega {\left[\mathrm{Ti}\right]}_{\%}\omega {\left[N\right]}_{\%})=\frac{\mathrm{lg}e\cdot \Delta G^{Ө}}{RT}+1.827$$

The calculation results of the JMatPro7.0 show that the solidus and liquidus temperatures of the IN718 alloy are 1360.15 K and 1640.95 K, respectively. By substituting *T_L_* and *T_S_* into Equation (7), the equilibrium concentration products of Ti and N required for TiN precipitation at both temperatures are obtained as follows:

(8) $$\omega {\left[\mathrm{Ti}\right]}_{\%}\omega {\left[N\right]}_{\%}=0.0035(T_{L}=1640.95K)$$

(9) $$\omega {\left[\mathrm{Ti}\right]}_{\%}\omega {\left[N\right]}_{\%}=0.000011(T_{S}=1360.15K)$$

**Figure 4:**
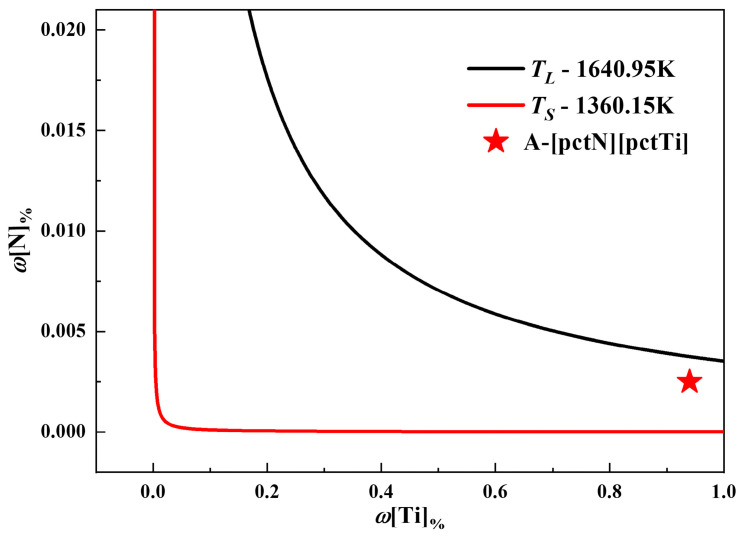
Stability curve of TiN inclusion precipitation.

A stability curve of TiN inclusion precipitation in the IN718 ingot was constructed from Equations (8) and (9), as shown in Figure 4. In this figure, the black and red lines represent the equilibrium curves of TiN precipitation of the molten alloy at the liquidus temperature and the solidus temperature, respectively, while point A denotes the actual concentration product of Ti and N. The data showed that the actual concentration product of Ti and N in IN718 exceeded the equilibrium concentration product at the solidus temperature but remained below it at the liquidus temperature, indicating that TiN inclusions precipitated during solidification. In fact, due to the differences in solubility of solute atoms between the solid and liquid phases, solute elements tend to accumulate at the solidification front. This elemental segregation behavior leads to an increase in the actual concentration product of Ti and N, ultimately causing TiN inclusions to precipitate earlier during the solidification process. Therefore, accurately evaluating the actual solute concentration at the solidification front is essential.

### 2.3. Microsegregation Models for Solute Elements

#### 2.3.1. Mathematical Models

The catastrophic influence of solute redistribution on microstructural homogeneity was realized decades ago for its ability to cause non-equilibrium phase, cracks, and other problems. Several models were developed to characterize the behaviors of these microsegregation elements. Based on the Lever Rule, the initial model assumes complete diffusion to the equilibrium of all solutes in both the liquid and the solid phase, which may be written as the following:

(10) $$\frac{C_{L}}{C_{0}}=\frac{1}{\left[1-(1-k)f_{S}\right]}$$

where *C_L_* is the concentration of the solute element concerned in the liquid part at the solidification front; *C*_0_ is the initial concentration before solidifying; *f_S_* is the solid fraction; and *k* is the equilibrium partition coefficient for the given element. The validation of this model is problematic due to the slow diffusion rate in the solid phase for large solute atoms.

The Scheil equation [22], the opposite limiting case to the L-R model, assumes complete diffusion in the liquid phase, no diffusion in the solid phase, and local equilibrium at the solid–liquid interface. This nonequilibrium model is the following:

(11) $$\frac{C_{L}}{C_{0}}={\left(1-f_{S}\right)}^{\left(k-1\right)}$$

This model works well at the initial stage of solidification, while it turns out to be a ‘disaster’ as *C_L_* approaches infinite at *f_S_* → 1. Hence, only when the cooling rates exceed 10 °C/s can this equation be applied.

Many simple models were proposed to predict microsegregation during steel solidification in view of finite nonzero diffusion in the solid phase. With fixed dendrite arm spacing taken into account, Brody and Fleming [9] proposed a general model. Ohnaka [11] and Clyne and Kurz [10] modified the back-diffusion parameters in this model. This equation leads to the following:

(12) $$\frac{C_{L}}{C_{0}}={\left[1-(1-2\Omega k)f_{S}\right]}^{\frac{\left(k-1\right)}{\left(1-2\Omega k\right)}}$$

(13) $$\Omega =\alpha \left[1-\exp\left(-\frac{1}{\alpha }\right)\right]-\frac{1}{2}\exp\left(-\frac{1}{2\alpha }\right)$$

(14) $$\alpha =\frac{4D_{S}t_{f}}{{\left(\lambda_{S}/10000\right)}^{2}}$$

where *α* is a Fourier number; *D_S_* is the diffusion coefficient of solute in the solid phase; *t_f_* is the local solidification time (LST); and *λ_S_* is the secondary dendrite arm spacing (SDAS). *Ω* tends to *α* as *α* → 0, and the C-K Equation (12) tends to the Scheil Equation (11). *Ω* tends to 1/2 as *α* → ∞, and the C-K Equation (12) tends to the ‘L-R’ Equation (10). The introduction of these parameters, i.e., *D_S_*, *t_f_*, and *λ_S_*, covers all rates of solute diffusion in the solid phase, which means a broad range of applicability. In the present work, Equations (12), (14), (17) and (18) are regarded as the basis of calculations.

More advanced solutions and models [23,24,25,26,27,28] for microsegregation were developed for high accuracy and board adaptability at the expense of accurate solidification process parameters, etc. Considering the effect of coarsening on the dilution of the liquid phase, Voller and Beckermann [12] added an additional back-diffusion term to the Fourier number, as follows:

(15) $$\alpha^{+}=\alpha +\alpha^{C}$$

This model could match full coarsening model results as *α^C^* = 0.1, which extended the model from plate to columnar dendrites. Columnar crystals are the major form of dendritic growth during the VAR process; consequently, this model also has a certain basis for the solidification process in VAR. As a result, Equation (13) changes into the following:

(16) $$\Omega =\alpha^{+}\left[1-\exp\left(-\frac{1}{\alpha^{+}}\right)\right]-\frac{1}{2}\exp\left(-\frac{1}{2\alpha^{+}}\right)$$

Based on the C-K model, Won and Thomas accepted the proposals of Ohnaka and Voller and Beckermann, considering the influence of columnar dendrites and coarsening on the back diffusion coefficient:

(17) $$\Omega =\alpha^{+}\left[1-\exp\left(-\frac{1}{\alpha^{+}}\right)\right]-\frac{1}{2}\exp\left(-\frac{1}{2\alpha^{+}}\right)$$

(18) $$\alpha^{+}=2(\alpha +\alpha^{C})$$

#### 2.3.2. Calculation of T_S-L_ During Solidification of the IN718

Previous thermodynamic theoretical works showed that the temperature at the solidification front of the molten alloy (*T_S-L_*) is a function of the liquidus temperature (*T_L_*), solidus temperature (*T_S_*), and solid-phase fraction (*f_S_*) [29]. This relationship is expressed as follows:

(19) $$T_{S-L}=T_{0}-\frac{T_{0}-T_{L}}{1-f_{S}\frac{T_{L}-T_{S}}{T_{0}-T_{S}}}$$

where *T*_0_ denotes the melting point of pure nickel, 1728.15 K; *T_L_* and *T_S_* are the liquidus and solidus temperatures, respectively (determined via JMatPro7.0 software), K; *T_S-L_* is the temperature of solidification front, K; and *f_S_* represents the solid fraction.

**Figure 5:**
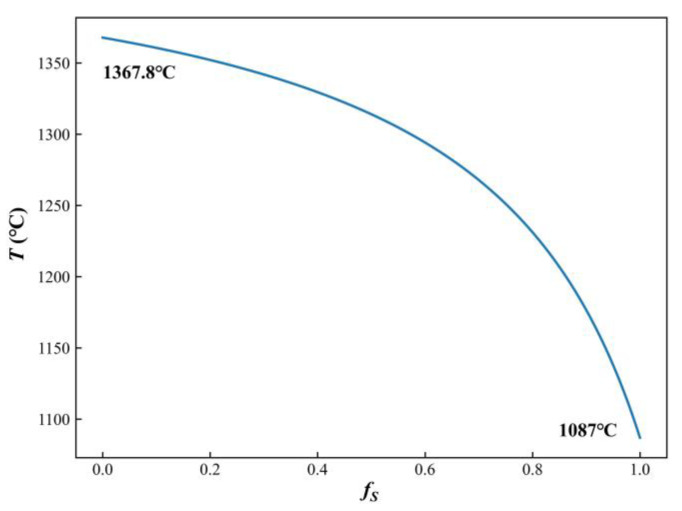
Curve of temperature at the solidification front as a function of solid fraction.

Figure 5 illustrates the relationship between the temperature at the solidification front (*T_S-L_*) and the solid fraction (*f_S_*). The results showed that solidification of the alloy began at 1367.8 °C when the solid fraction was zero. As the temperature decreased, the solid fraction in the alloy gradually increased, with the solidification rate accelerating until full solidification was reached at 1087 °C, where the solid fraction was 1.

Table 3 provides data on equilibrium partition coefficients and the diffusivity of solute elements in γ iron, showing that diffusion coefficients of solute elements vary with temperature. This study incorporated the temperature dependence of diffusivity into the model for accurate analysis.

#### 2.3.3. Calculation of Cooling Rate

To proceed with the thermodynamic calculations, the value of *C_L_* as *f_S_* increases is essential. In Equation (12), *C*_0_ and *k* are constants, indicating that *Ω* is a key parameter. The local solidification time is defined in Equation (14) as follows:

(20) $$t_{f}=\frac{T_{L}-T_{S}}{C_{R}}$$

where *T_L_* and *T_S_* are the liquidus and solidus temperatures of the alloy (determined via JMatPro7.0 software), K; and *C_R_* is the cooling rate, °C/min.

Previous experiments measured the secondary dendrite arm spacing of the IN718 alloy under various cooling rates and established a functional relationship between secondary dendrite arm spacing and the cooling rate [31]. However, errors were identified in the original fitted results, which prompted a re-fitting in this study, yielding the following:

(21) $$\lambda_{S}=183.72\times {C_{R}}^{-\frac{1}{3}}-13.749$$

where *λ_S_* represents the secondary dendrite arm spacing of the IN718 alloy, μm.

Based on the theoretical foundation of Equation (21), the local cooling rate within the alloy ingot could be estimated by measuring the secondary dendrite arm spacing. In this study, Equation (21) was incorporated into a program that calculates and outputs the cooling rate when the secondary dendrite arm spacing was provided. Additionally, the local solidification time (*t_f_*) was determined by substituting the calculated cooling rate into Equation (20).

**Figure 6:**
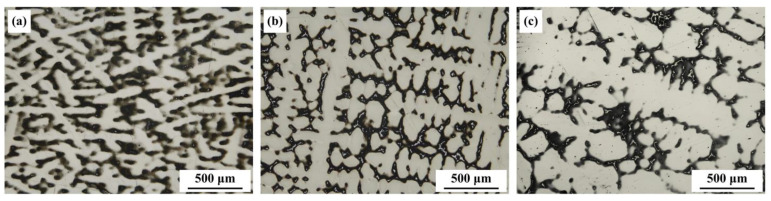
Dendrite morphology corresponding to different secondary dendrite arm spacing in solidification microstructure: (**a**) 72 μm; (**b**) 108 μm; and (**c**) 138 μm.

Due to variations in water flow rate and cooling conditions from the outer to the inner regions, the secondary dendrite arm spacing in the ingot’s solidification structure ranged from 72 to 138 μm. Figure 6 displays dendrite morphologies corresponding to typical secondary dendrite arm spacing values. The figure shows that dendrites coarsen progressively as secondary dendrite arm spacing increases. To assess the impact of different cooling rates on microsegregation and TiN precipitation, three representative secondary dendrite arm spacing values (70 μm, 105 μm, and 140 μm) were selected for further analysis.

### 2.4. Calculation of TiN Precipitation

#### 2.4.1. Microsegregation Calculations of Solute Elements

During solidification, microsegregation caused the concentrations of N and Ti in the residual liquid phase at the solidification front to increase as the solid fraction grew. TiN inclusions began to precipitate when the actual concentration product of Ti and N in the residual melt exceeded the equilibrium solubility product of TiN, resulting in the consumption of N and Ti in the liquid phase.

In a fully coupled model, the consumption of both N and Ti during TiN precipitation should be considered [32]. However, in this study, with an initial N concentration of only 0.0025%, it was assumed that all N was consumed during TiN precipitation, while only 0.0085% of Ti was used. Given that the Ti content in the IN718 alloy was approximately 0.94%, which significantly exceeded the amount that could be consumed by TiN precipitation, this model neglected Ti consumption and focused solely on N consumption.

Before TiN precipitation, the concentration of each alloying elements was calculated using Equation (22):

(22) $$\frac{C_{L}}{C_{0}}={\left[1-(1-2\Omega k)f_{S}\right]}^{\frac{\left(k-1\right)}{\left(1-2\Omega k\right)}}$$

After TiN precipitation, the concentrations of Ti and other elements continued to be calculated using the same equation, but with adjustments to the N concentration in the residual liquid phase. The N concentration at step *n* was determined using Equation (23):

(23) $$C_{L,n}=C_{L,\mathrm{eq},n-1}\frac{{\left[1-(1-2\Omega_{n}k_{N})f_{S}\right]}^{\frac{\left(k_{N}-1\right)}{\left(1-2\Omega_{n}k_{N}\right)}}}{{\left[1-(1-2\Omega_{n-1}k_{N})(f_{S}-\Delta f_{S})\right]}^{\frac{\left(k_{N}-1\right)}{\left(1-2\Omega_{n-1}k_{N}\right)}}}$$

where *C_L,n_* is the N concentration in the calculation cell at step, wt.%; *C_L_*_,eq,*n*−1_ is the equilibrium N concentration in the calculation cell at step (*n* − 1), wt.%; *Ω_n_*_−1_ and *Ω_n_* are the *Ω* parameter values for steps *n* − 1 and *n*, respectively; and *k*_N_ is the equilibrium partition coefficient of N between the solid and liquid phases.

#### 2.4.2. Effect of Microsegregation of Other Alloying Elements on TiN Precipitation

During solidification, microsegregation behavior is common among all alloying elements. The enrichment of these elements in the residual liquid phase alters the activity coefficients of N and Ti, thereby influencing TiN precipitation. Consequently, the program developed for this study accounted for the microsegregation effects of six key alloying elements: C, S, Nb, Mg, Al, and O. The software was equipped with a flexible interface, which enabled the seamless integration of additional equilibrium partition coefficients and diffusion coefficients for alloying elements as they became available.

### 2.5. The Unit and Process of Calculation

**Figure 7:**
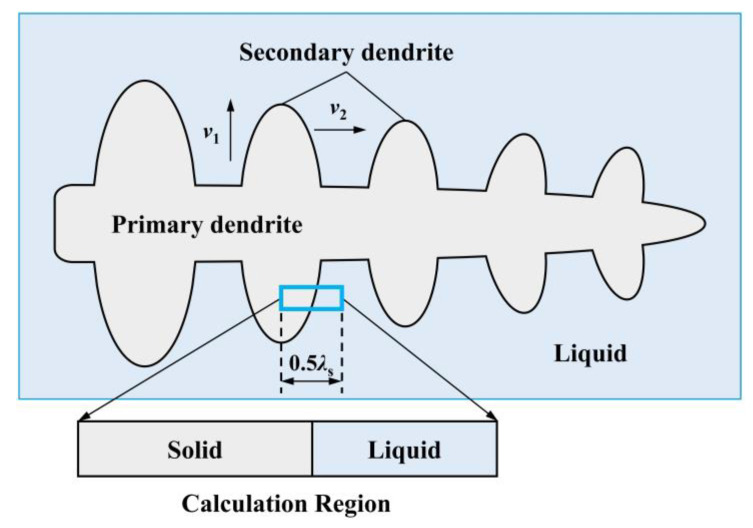
Schematic of the solidification calculation unit.

During solidification, primary dendrites initiate growth, with secondary dendrites forming symmetrically on either side of the primary dendrites due to the temperature gradient at the solidification front. Simultaneously, the solid–liquid interface on the secondary dendrites gradually advances toward the center of the interdendritic region. As shown in Figure 7, the computational unit of the coupling model used in this study started at the central axis of a secondary dendrite in the mushy zone and extended to half the length of the secondary dendrite arm spacing.

**Figure 8:**
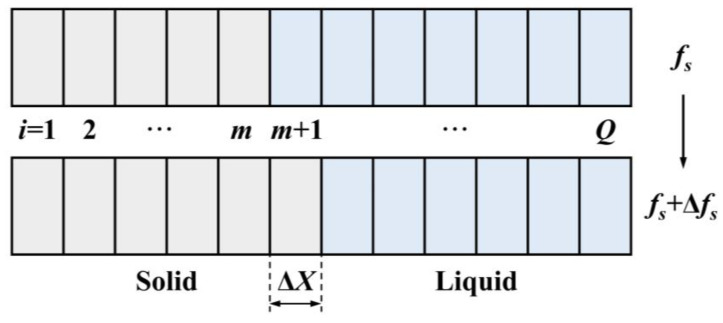
Schematic of the division of the calculation region.

The solidification region, defined as half of the secondary dendrite arm spacing (*λ_S_*), was divided into *Q* segments, each with a length of Δ*X*, as shown in Figure 8. The calculation step size was Δ*f_S_*. For each computational step, the solid fraction increased from *f_S_* to *f_S_ +* Δ*f_S_*, advancing the solidification front by one unit and increased the number of solid phase cells from *m* to *m* + 1.

Before starting the calculations used the coupled model, the following assumptions were made:

(1) Mass transfer in the calculation region was governed solely by diffusion. Solute diffusion was limited in the solid phase but remained uniform in the liquid phase.
(2) Dendrite growth followed a parabolic shape, as shown in Figure 7.
(3) The solid–liquid interface was treated as a planar surface.
(4) At the solidification front, the reaction between Ti and N in the liquid phase reached equilibrium, satisfying the relationship in Equation (4).
(5) The calculated unit represented half of the secondary dendrite arm spacing, as shown in Figure 7.
(6) The precipitation of other types of inclusions during solidification was disregarded.

**Figure 9:**
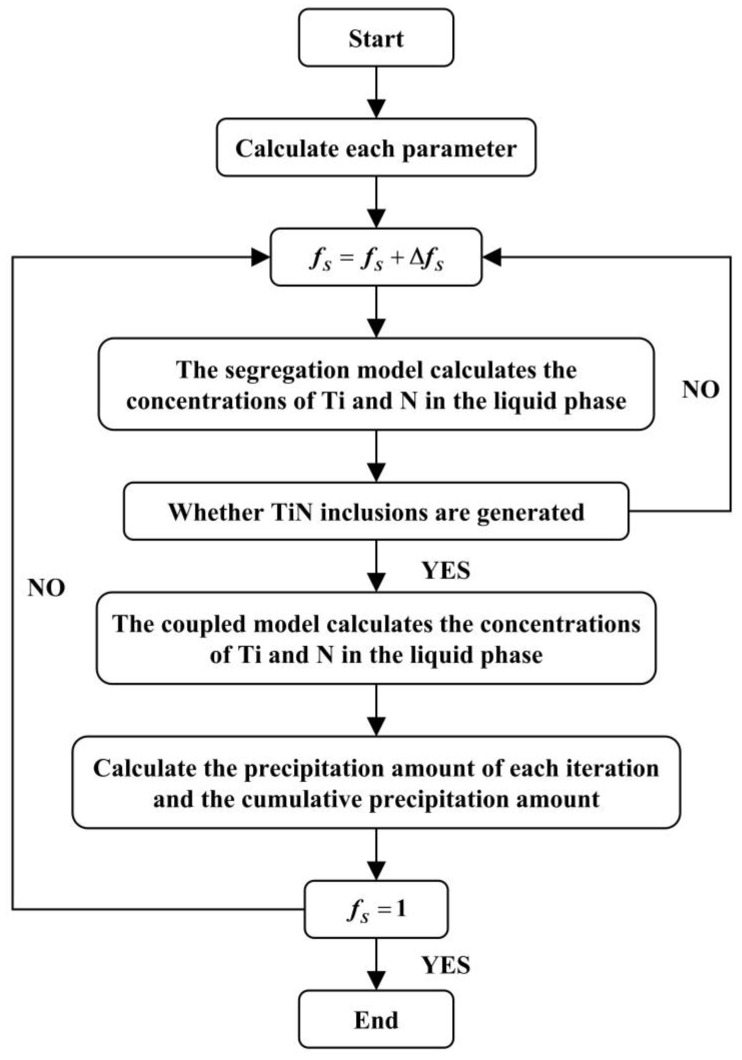
The main calculation process of the model.

The overall calculation process of the coupled model was depicted in Figure 9. In each iteration step, *f_S_* was incremented by Δ*f_S_* (with *f_S_* = 0 for the initial step). First, the concentrations of Ti and N in the molten alloy were calculated using the modified C-K model, followed by an evaluation using Equation (2) to determine whether TiN inclusions precipitated. If no precipitation occurred, the process moved to the next step. If TiN precipitation was detected, the microsegregation module was coupled with the TiN precipitation module to compute the concentrations of Ti and N in the residual liquid phase. Finally, the TiN precipitation amount and its cumulative value were calculated for each step. The process continued until *f_S_* = 1, marking the end of the calculation.

## 3. Results and Discussion

### 3.1. Discussion of Fundamental Results

#### 3.1.1. Thermodynamic Calculation Results

**Figure 10:**
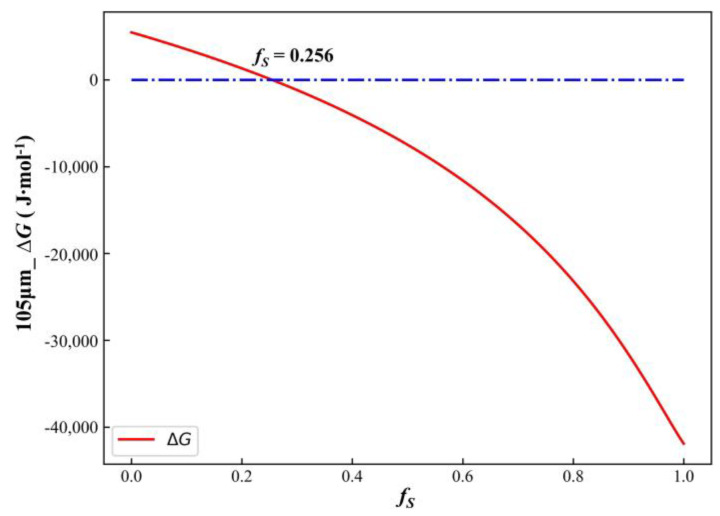
The Δ*G* change curve of the TiN precipitation reaction during solidification.

Using Equations (1)–(3), the Δ*G* for the precipitation reaction of TiN inclusions at different solid fractions was calculated to determine whether TiN precipitated. Using a secondary dendrite arm spacing of 105 μm as an example, Figure 10 shows the variation in the Gibbs free energy (Δ*G*) during solidification. The graph indicated that, as solidification progressed, the solid fraction (*f_S_*) increased and Δ*G* decreased. For 0 ≤ *f_S_* < 0.256, Δ*G* > 0, indicating that no TiN precipitation occurred in the residual liquid phase. At *f_S_* = 0.256, Δ*G* = 0, marked the critical point for TiN precipitation. Substituting *f_S_* into Equation (19) yielded a critical temperature for TiN precipitation at the solidification front of 1619.81 °C. For 0.256 < *f_S_* ≤ 1, Δ*G* < 0, indicating the onset of TiN precipitation, which continued to increase as *f_S_* rose. The maximum amount of TiN precipitation was reached when solidification was complete (*f_S_* = 1). Reducing the initial concentrations of N and Ti in the molten alloy effectively lowered the free energy of the TiN precipitation reaction, delaying the threshold for TiN formation. This adjustment also decreased the total amount of TiN precipitated at the end of solidification and reduced the potential for TiN inclusion agglomeration.

#### 3.1.2. Calculation Results of the Coupled Model

**Figure 11:**
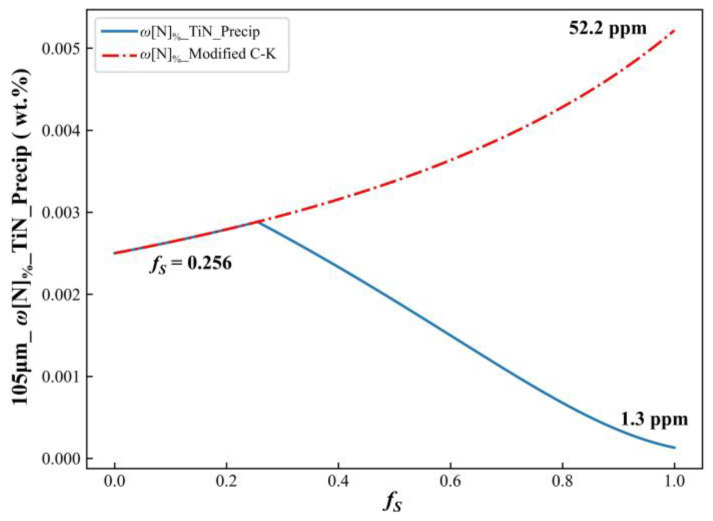
N concentration curves in the residual liquid phase were predicted using the modified C-K model and the coupled model, respectively.

By accounting for the consumption of N due to TiN precipitation (through the coupling of the microsegregation module with the TiN precipitation module), the variation in N concentration in the residual liquid phase under coupled conditions was obtained. Using a secondary dendrite arm spacing of 105 μm as an example, the computational results are shown in Figure 11. The results indicated that, without considering the impact of TiN precipitation, the N concentration in the residual liquid phase gradually increased during solidification, reached 52.2 ppm by the end of the process. When the effects of both microsegregation and TiN precipitation on the N concentration were included, the results were as follows: for *f_S_* < 0.256, the outputs from the coupled model and the microsegregation model alone were identical. At *f_S_* = 0.256, TiN precipitation began in the residual liquid phase. For *f_S_* > 0.256, as solidification progressed, the N concentration in the residual liquid phase decreased, and the extent of N microsegregation diminished. By the end of solidification, the N concentration dropped to just 1.3 ppm, indicating that nearly all N precipitated as primary nitride or carbonitride during the solidification process.

#### 3.1.3. Microsegregation of Other Alloying Elements

**Figure 12:**
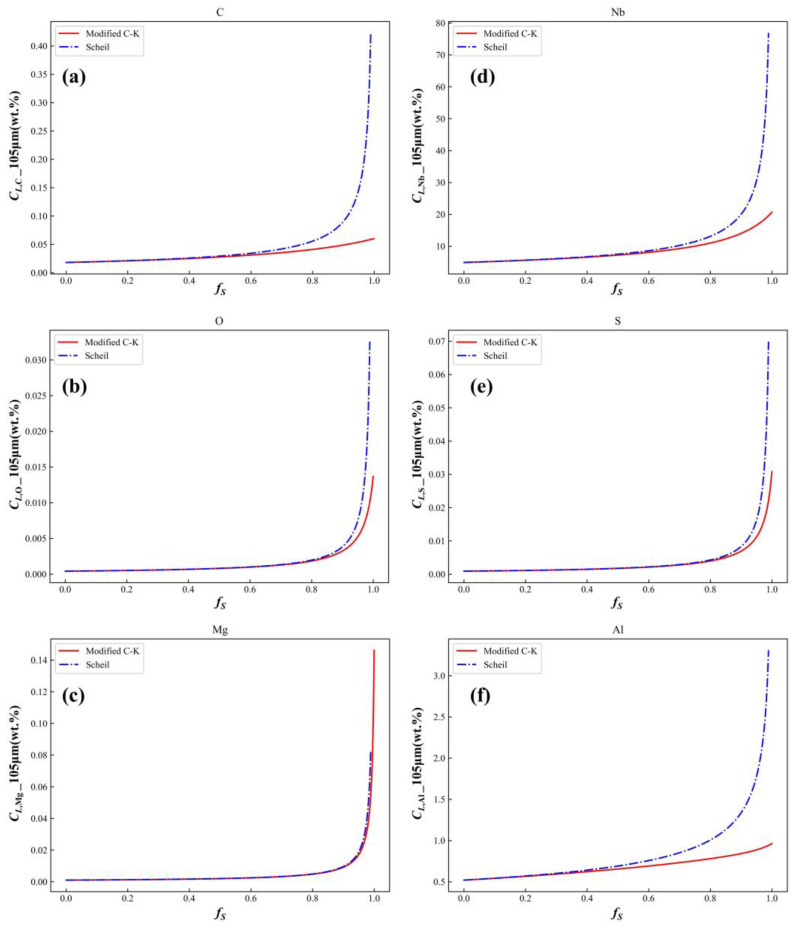
Microsegregation of the six major alloying elements calculated by the Scheil model and the modified C-K model: (**a**) C; (**b**) O; (**c**) Mg; (**d**) Nb; (**e**) S; (**f**) Al.

The influence of microsegregation of major alloying elements on TiN precipitation was examined. Using a secondary dendrite arm spacing of 105 μm as an example, the microsegregation of six key elements—C, O, Mg, Nb, S, and Al—was predicted using both the modified C-K model and the Scheil model. The results are shown in Figure 12. The microsegregation data for Ti and N were excluded from this comparison, as their behavior was influenced by TiN precipitation.

Figure 12 shows the concentration change curves for six major alloying elements calculated by the Scheil model and the modified C-K model. These curves reveal that, regardless of whether the modified C-K model or the Scheil model is used, the concentration of each alloying element in the residual liquid phase increases as solidification progresses, with a marked acceleration in the later stages. During the initial stages of solidification, the concentrations calculated by the Scheil model closely align with those obtained using the modified C-K model. However, in the later stages, the Scheil model predicts much higher concentrations than the modified C-K model, which does not align with physical expectations. Table 4 compares the microsegregation ratios of these six elements as calculated by the modified C-K model. The data indicated that the order of microsegregation ratios during solidification was Mg > S > O > Nb > C > Al. Notably, the microsegregation of Mg and S became particularly pronounced as solidification advanced, highlighting their significant impact on TiN precipitation.

#### 3.1.4. Effect of Different Models

**Figure 13:**
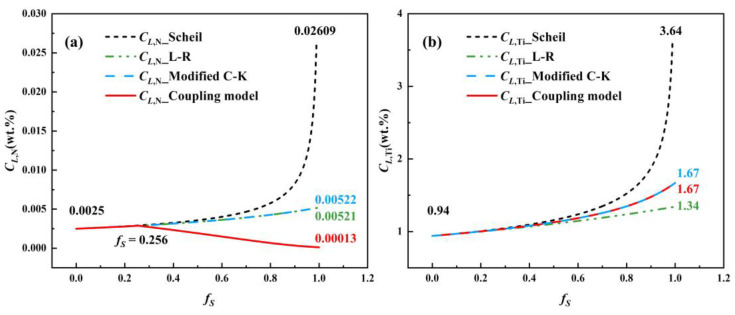
Curves of Ti and N concentrations predicted by different microsegregation models: (**a**) N; and (**b**) Ti.

The microsegregation behavior of Ti and N was analyzed using the L-R model, Scheil model, modified C-K model, and the coupled model, with a secondary dendrite arm spacing of 105 μm. For accuracy, the results for *f_S_* > 0.99 were excluded due to the Scheil model’s limitations at the final stage of solidification. Figure 13 presents the concentration variation curves for Ti and N.

Figure 13a,b show that, at the start of solidification, the initial concentrations of N and Ti were 0.0025 wt.% (25 ppm) and 0.94 wt.%, respectively. Before TiN precipitation (*f_S_* < 0.256), the concentration results for Ti and N in the residual liquid phase were nearly identical across all models.

However, after TiN precipitation began, the behavior of N diverged. The L-R, Scheil, and modified C-K models predicted increasing N concentrations as solidification progressed, whereas the coupled model showed a decreasing trend due to N consumption by TiN precipitation. The highest N concentration at the end of solidification was 0.02609 wt.% (260.9 ppm), as predicted by the Scheil model. The L-R model and modified C-K model provided similar results, at 0.00521 wt.% (52.1 ppm) and 0.00522 wt.% (52.2 ppm), respectively, between the Scheil and coupled model. The coupled model yielded the lowest N concentration, 0.00013 wt.% (1.3 ppm), reflecting the significant N consumption associated with TiN precipitation. The modified C-K model’s results closely resembled those of the L-R model due to the N element diffusing faster in the solid phase.

For Ti, all four models predicted an increasing concentration as solidification advanced. The Scheil model forecasted the highest Ti concentration at 3.64 wt.% at the end of solidification. The coupled model and modified C-K model produced similar results of 1.67 wt.%, intermediate between the predictions of the Scheil and L-R models, because the effect of TiN precipitation on Ti concentration was not considered. The L-R model predicted the lowest Ti concentration, at 1.34 wt.%.

#### 3.1.5. Effects of Coupled Model

**Figure 14:**
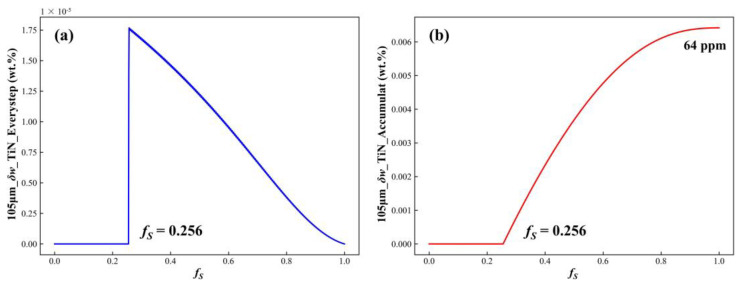
(**a**) TiN precipitation at each step; (**b**) accumulated TiN precipitation during solidification.

Using a secondary dendrite arm spacing of 105 μm and a step size of 0.001 as an example, the TiN precipitation amount at each step and the total accumulated precipitation were calculated. The variation curves for stepwise precipitation and accumulated precipitation as functions of the solid fraction are shown in Figure 14. Figure 14a indicates that, when *f_S_* < 0.256, the concentration product of Ti and N in the residual liquid phase is below the equilibrium concentration product, resulting in no TiN precipitation. Once *f_S_* ≥ 0.256, TiN begins to precipitate in the residual liquid phase. As the solid fraction increases, the amount of TiN precipitated at each step gradually decreases until it reaches zero at the end of solidification. Figure 14b illustrates that, when *f_S_* < 0.256, there is no accumulation of TiN in the residual liquid phase. When *f_S_* ≥ 0.256, the accumulated amount of TiN increases progressively with the solid fraction. However, the rate of accumulation slows as solidification continues, eventually reaching zero at the end of solidification. By the completion of solidification, the total accumulated TiN precipitation reaches 64 ppm.

### 3.2. Effect of Initial Concentration of N on TiN Precipitation

**Figure 15:**
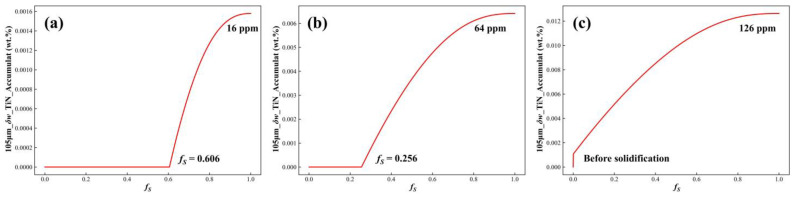
Curves of TiN accumulated precipitation with solid fraction under different initial N concentrations: (**a**) 10 ppm; (**b**) 25 ppm; and (**c**) 40 ppm.

Using a secondary dendrite arm spacing of 105 μm as an example, the accumulated precipitation amounts of TiN in the residual liquid phase were calculated for initial N concentrations of 10, 25, and 40 ppm. The variation curves of accumulated TiN precipitation as a function of the solid fraction are shown in Figure 15. The results indicated that, as the initial N concentration increased, TiN began to precipitate earlier, and the accumulated precipitation at the end of solidification rose significantly. At an initial N concentration of 10 ppm, TiN started to precipitate at *f_S_* = 0.606. As solidification progressed, the precipitation gradually increased, reaching an accumulated amount of 16 ppm by the end of solidification. When the initial N concentration was raised to 25 ppm, TiN began to precipitate at *f_S_* = 0.256, and the accumulated precipitation at the end of solidification increased notably to 64 ppm. At an initial N concentration of 40 ppm, TiN precipitation started before solidification began, and the final accumulated precipitation reached 126 ppm. The results indicated that higher initial nitrogen concentrations led to earlier TiN precipitation and a greater total amount of TiN formed. The increased number of TiN particles provided abundant heterogeneous nucleation sites for NbC precipitation. During the later stages of solidification, these TiN and NbC particles further evolved into mixed (Nb + Ti) carbonitrides. These precipitates exhibited poor distribution uniformity within the structure and were difficult to adjust effectively through subsequent heat treatment or homogenization, ultimately becoming part of the solidified structure and negatively impacting the mechanical properties of the alloy [7]. Therefore, controlling the nitrogen concentration in the alloy was crucial for optimizing the microstructure and enhancing the mechanical properties of the alloy.

### 3.3. Effect of Cooling Rate on TiN Precipitation

**Figure 16:**
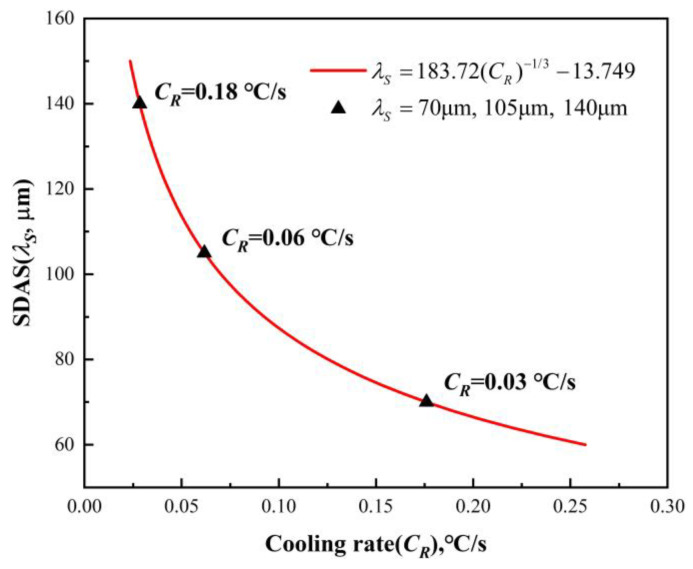
Curve of secondary dendrite arm spacing at different cooling rates.

The curve of the secondary dendrite arm spacing against the cooling rate was established based on the re-fitting Equation (21). The local cooling rates corresponding to secondary dendrite arm spacings of 70 μm, 105 μm, and 140 μm were predicted. The results are shown in Figure 16. This figure shows that the secondary dendrite arm spacing gradually decreases as the cooling rate increases.

**Figure 17:**
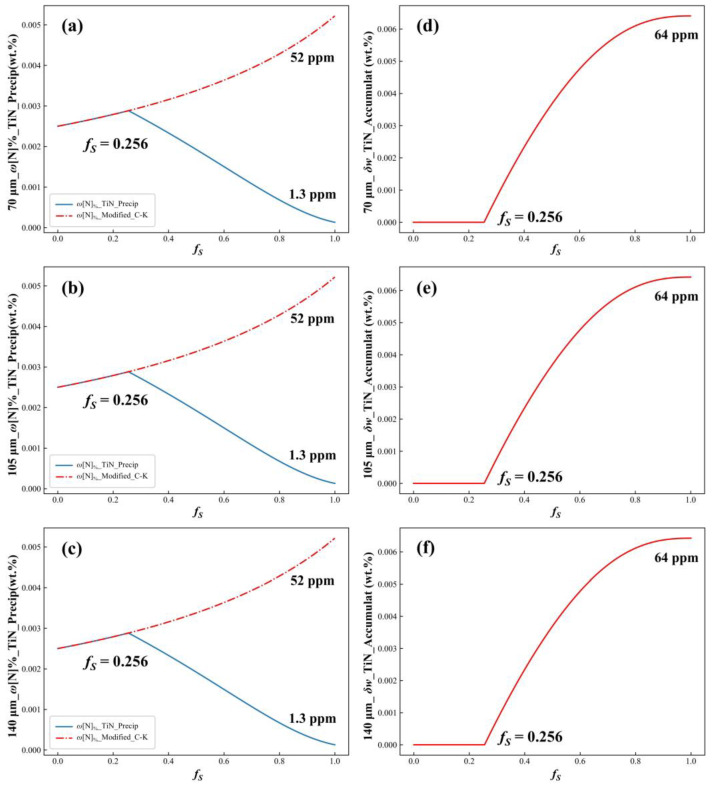
The concentration of N and TiN accumulated precipitation in the residual liquid phase during solidification at different cooling rates: (**a**,**d**) *C_R_* = 0.18 °C/s; (**b**,**e**) *C_R_* = 0.06 °C/s; (**c**,**f**) *C_R_* = 0.03 °C/s.

For secondary dendrite arm spacing values of 70 μm, 105 μm, and 140 μm, the corresponding local cooling rates were 0.18 °C/s, 0.06 °C/s, and 0.03 °C/s, respectively. Using the coupled model, the N concentration in the residual liquid phase and the accumulated TiN precipitation at these cooling rates were estimated, with the results shown in Figure 17. This figure revealed that increasing the cooling rate from 0.03 °C/s to 0.18 °C/s had few effects on the N concentration and accumulated TiN precipitation. When the impact of TiN precipitation on the N concentration was ignored, the N concentration at the end of solidification remained 52 ppm for all three cooling rates. When TiN precipitation was considered, the final N concentration dropped to 1.3 ppm for all rates, with a total of 64 ppm of TiN precipitation throughout the solidification process. These findings suggested that increasing the cooling rate had little effect on solute microsegregation or TiN precipitation within dendritic cells. However, higher cooling rates effectively reduced secondary dendrite arm spacing, thereby decreasing the size of microsegregation units (half of the secondary dendrite arm spacing), which significantly influenced solute microsegregation and TiN precipitation.

## 4. Conclusions

Large TiN inclusions in nickel-based superalloys promote micropore formation, compromising the mechanical properties of the alloys. However, current research lacks a comprehensive coupled model that considers both solute element microsegregation and TiN precipitation specifically in nickel-based superalloys. This study integrated microsegregation and TiN precipitation calculations to conduct a comprehensive thermodynamic analysis of element microsegregation and TiN precipitation during the solidification of IN718 alloys. The effects of the coupled model, multi-element microsegregation, initial N concentration, and the cooling rate on TiN precipitation were evaluated. The main conclusions are as follows:

1. The microsegregation ratios of solute elements (excluding Ti and N) in the IN718 molten alloy followed the order: Mg > S > O > Nb > C > Al. Niobium showed the most significant increase in concentration during solidification (approximately 15.75 wt.%), due to its high initial concentration. This finding highlights the importance of considering multi-element microsegregation in thermodynamic calculations of TiN precipitation in the IN718 alloy.
2. Without coupling the TiN precipitation module with the microsegregation module, the N concentration in the residual liquid phase rose to 52.2 ppm by the end of solidification. When the modules were coupled, the N concentration decreased to 1.3 ppm after TiN precipitation, demonstrating a marked difference between the two scenarios.
3. Thermodynamic analysis indicated that TiN inclusions began to precipitate when the solid fraction exceeded 0.256. As solidification progressed, the amount of TiN precipitation increased and reached 64 ppm by the end of solidification.
4. With an increase in the initial N concentration from 10 ppm to 40 ppm, the solid fraction at which TiN precipitation began shifted from 0.606 to a point before the onset of solidification, and the accumulated TiN precipitation during solidification increased from 16 ppm to 126 ppm.
5. The variation in N concentration and accumulated TiN precipitation was predicted for cooling rates of 0.03 °C/s, 0.06 °C/s, and 0.18 °C/s. The results were nearly identical, suggesting that changes in cooling rate had little effect on TiN precipitation.

## Figures and Tables

**Table 1:** Chemical composition of the IN718 alloy (wt.%).

C	Si	Mn	P	S	Cr	Fe	Cu
0.018	0.05	0.01	0.003	0.0009	17.88	18.73	0.01
**Mo**	**Ti**	**Nb**	**Mg**	**Al**	**B**	**Co**	**Pb**
2.96	0.94	4.91	0.001	0.52	0.0032	0.01	0.0001
**Bi**	**Ca**	**Se**	**O**	**N**	**H**	**Ni**	
0.00005	0.001	0.0005	0.00041	0.0025	0.00003	Bal.	

**Table 2:** Activity interaction coefficient of components in molten nickel (1873 K).

	Element	C	Si	Mn	P	S	Cr	Cu	Mo	Ti	Nb
$e_{i}^{j}$	N	0.09	0.05	−0.05	0.05	0.01	−0.08	0.01	−0.04	−0.21	−0.07
	Ti	0	0	0	0	0	0.025	0	0	0.048	0
$r_{i}^{j}$	N	0	0	0	0	0	0	0	0	0	0
	Ti	0	0	0	0	0	0	0	0	−0.001	0
	**Element**	**Al**	**B**	**Co**	**O**	**N**	**Ta**	**Fe**	**W**	**V**	**Zr**
$e_{i}^{j}$	N	0	0.09	−0.01	0.05	0	−0.07	−0.01	−0.02	−0.13	−0.23
	Ti	0.0037	0	0	−1.8	−2.041	0	0	0	0	0
$r_{i}^{j}$	N	0	0	0	0	0	0	0	0	0	0
	Ti	0	0	0	0	0	0	0	0	0	0

**Table 3:** Equilibrium partition coefficients (*ks*) and diffusion coefficients (*D_S_*) of solute elements in γ phase [30].

Element	*k*	*D_S_* (m^2^/s)
C	0.3	0.000015 Exp (−143,511/R*T*)
S	0.035	0.00024 Exp (−223,426/R*T*)
Ti	0.7	0.000015 Exp (−250,956/R*T*)
Nb	0.39	0.000083 Exp (−266,479/R*T*)
Mg	0.02	0.0000055 Exp (−249,366/R*T*)
Al	0.59	0.00059 Exp (−241,417/R*T*)
O	0.03	0.000575 Exp (−168,615/R*T*)
N	0.48	0.000091 Exp (−168,490/R*T*)

**Table 4:** The results of microsegregation of six major alloying elements were calculated using the modified C-K model.

	Initial Concentration *C*_0_ (wt.%)	Terminal Concentration *C_L_* (wt.%)	Concentration Gradient Δ*C* (wt.%)	Microsegregation Ratio Δ*C*/*C*_0_
C	0.018	0.060	0.042	2.3
S	0.0009	0.0308	0.0299	33.2
Nb	4.91	20.66	15.75	3.2
Mg	0.001	0.146	0.145	145.0
Al	0.52	0.96	0.44	0.8
O	0.00041	0.01368	0.01327	32.4

## Data Availability

The original contributions presented in the study are included in the article, further inquiries can be directed to the corresponding author.
